# The use of Resuscitative Endovascular Balloon Occlusion of the Aorta in a pregnant woman with a ruptured splenic aneurysm and haemorrhagic shock: A case report

**DOI:** 10.1111/aas.14172

**Published:** 2022-11-16

**Authors:** Marte Irene Skille Carlsen, Nina Kirksæther Aalberg, Alina Desiree Sandø, Solveig Skrede, Sven Erik Gisvold, Oddvar Uleberg

**Affiliations:** ^1^ Department of Anaesthesia and Intensive Care St. Olav's University Hospital Trondheim Norway; ^2^ Department of Radiology and Nuclear Medicine St. Olav's University Hospital Trondheim Norway; ^3^ Department of Surgery St. Olav's University Hospital Trondheim Norway; ^4^ Department Gynaecology and Obstetrics St. Olav's University Hospital Trondheim Norway; ^5^ Department of Circulation and Medical Imaging Norwegian University of Science and Technology Trondheim Norway; ^6^ Department of Emergency Medicine and Pre‐hospital services St. Olav's University Hospital Trondheim Norway

**Keywords:** haemorrhagic shock, hyperkalaemia, massive transfusion, pregnancy, REBOA, splenic artery aneurism

## 1 BACKGROUND

The earliest reported use of Resuscitative Endovascular Balloon Occlusion of the Aorta (REBOA) was during the Korean War in the 1950s.^1^ With the development of advanced endovascular techniques, REBOA has emerged as a less invasive alternative to aortic cross‐clamping for resuscitation and temporary haemorrhagic control.^2^ Contemporary studies also suggest that REBOA reduces mortality in trauma patients with sub‐diaphragmatic haemorrhagic shock,^3^ and is associated with improved survival in trauma patients with non‐compressible torso haemorrhage treated in a high‐volume centre.^4^ Still, the indications for REBOA remain poorly defined, and there is conflicting evidence regarding the safety and efficiency of the procedure.^5^ We present a case of splenic artery aneurism in a pregnant woman, where REBOA was introduced late and standard surgical procedures failed to prevail, but provided a vital bridge to definitive treatment. We suggest that this technique should be used more frequently and be on the repertoire for anesthesiologists.

## 2 CASE PRESENTATION

A 31‐year‐old healthy woman, 11 weeks pregnant, was admitted to the emergency department after an unwitnessed syncope. The clinical examination revealed a patient in severe hypotensive shock; pale, sweaty and confused. Her vitals included a GCS of 13, pulse 80/min and NIBP 52/31 mmHg. ABG showed a haemoglobin of 11.6 g/dl, pH 7.29, pCO_2_ 6.2 kPa, BE −8.6 mmol/L and lactate 5.5 mmol/L. We observed bradycardia relative to the patient's low blood pressure. This has previously been described in the setting of haemorrhagic shock, and is not an uncommon finding. The mechanism behind is suspected to be a parasympathetic reflex due to hemoperitoneum.^6^

Focused assessment with sonography showed a vital intrauterine pregnancy and substantial amounts of intra‐abdominal fluid. Following transfusion of two units of erythrocyte suspension (SAG‐M blood), the physiology improved significantly allowing for a CT scan. This confirmed an extensive hemoperitoneum without any contrast leakage. It also demonstrated a saccular aneurysm on the splenic artery (SAA) of 13 mm, but with no indication of ongoing bleeding. The aneurysm was considered a possible incidental finding with pregnancy‐related hemoperitoneum as the working diagnosis.

Post‐CT, the patient again became haemodynamically unstable and was brought to the operating room (OR). At the start of surgery, the systolic BP was 58 mmHg and with a pulse of 122/min. When unpacking it became clear that the ruptured SAA was the source. Splenectomy was first considered, but due to the anatomical location of the aneurysm partly dorsally to the pancreas and in the medial segment of the splenic artery, this would not provide haemorrhage control.

Despite re‐packing and efforts made to control and correct the metabolic changes, the patient was now deep into the lethal triad and still in need of a massive transfusion. Prioperatively the pulse followed an expected tachycardic pattern varying between 100–160/min. At the end of the surgery, the ABG showed a pH 7.07, pCO_2_ 5.6 kPa, BE −18.1 mmol/L and a lactate of 6.6 mmol/L. She also developed a hyperkalemia of 8 mmol/L, and with progressive ECG changes which was treated according to protocol.

As surgical procedures failed to prevail, endovascular treatment (REBOA) was now considered to be the best option in this situation. Both because of the immediate availability of an interventional radiologist to perform the procedure and as REBOA was considered a less invasive alternative to lateral thoracotomy and aortic cross‐clamping. This would require transportation to a nearby hybrid OR, and 90 min after admission, the decision to perform REBOA was made to ensure temporary haemorrhagic control during transit.

Using ultrasound guidance, the interventional radiologist inserted a 6 French 23 cm long introducer sheath percutaneously in the patient's common femoral artery. Over a J‐curved guidewire a 15 × 30 mm non‐compliant balloon was inserted 50 cm from the puncture site. This corresponded to the lower descending aorta—Zone 1. From insertion, the procedure took less than 2 min, resulting in an immediate response with systolic BP rising from 80 to 160 mmHg and a reduction in pulse rate from 144 to 77/min.

Transportation to the hybrid OR was completed without incident. Blood pressure remained stable around 130 mmHg until balloon deflation to allow for radiological intervention. Total balloon inflation time approximated 35 min. Definitive haemorrhagic control was achieved approximately 4 h after admission. The total amount of transfused blood products was 32 units of red blood cells (8000 ml), 29 units of plasma (5800 ml) and 9 units of thrombocyte platelets (2250 ml). Prior to ICU admission, her physiology had improved significantly and the ABG showed a pH 7.45, pCO_2_ 4.7 kPa, BE 0.3 mmol/L, lactate 4.1 mmol/L and a potassium of 3.8 mmol/L.

The patient was extubated the next day with no development of systemic inflammatory response syndrome, reperfusion ischaemia or further bleeding post‐operatively. There was no deterioration of liver or kidney function despite the prolonged inflation time, and there was no thrombosis or ischaemia related to the balloon or introducer sheath itself. The foetus was no longer vital.

## 3 DISCUSSION

Our patient was a young, pregnant woman in extremis due to a ruptured SAA. This resulted in massive transfusions and a resulting malignant hyperkalaemia. Given these complications, there was a need for a resuscitative bridge to definitive treatment, through either a lateral thoracotomy for external clamping of the aorta or placement of a REBOA.

In this situation, aortic occlusion proved vital. However, the REBOA was placed late into the event. The patient had been consistently unstable for a long time, with manifesting consequences of massive transfusion. Currently, there is still inconclusive evidence whether REBOA reduces the need for blood transfusions,^7^ although there are some emerging reports observing reduced transfusion needs.^8^, ^9^ We believe there is good reason to consider proactive use of REBOA,^10^ to reduce the dose of haemorrhagic shock and the need for massive transfusion with its associated risks.^11^

In this case, an interventional radiologist was needed to place the REBOA. However, it has been shown that anaesthesiologists, trained in the use of ultrasound and Seldinger technique, can master REBOA placement.^12^

There are known side effects of REBOA to keep in mind, such as reperfusion ischaemia, acute kidney injury, thrombosis and peripheral ischaemia.^13^ The CT‐scan showed that the patient's aortic diameter was 14 mm in the aorta descendens, and 12 mm in the infrarenal aorta (Zone 3). With a non‐compliant balloon of 15 mm, we must assume that the aorta descendens was fully occluded. Inflation of an oversized balloon in a fragile or slender aorta can have catastrophic consequences.^14^ As risk‐reducing measures, the procedure in our hospital consists of balloon placement over a wire through a 6 French 23 cm introducer. We also use a non‐compliant balloon with a maximum diameter of 15 mm, minimising the risk of aortic rupture. According to hospital standard operating procedure, the balloon should be deflated for approximately 1 min every 10–15 min to allow for perfusion of pelvic organs and lower extremities when the balloon is located in aortic Zone 3. We intend to deflate the balloon in the same manner when placing it in Zone 1 to avoid renal and visceral ischaemia. This was not done due to the critical state of the patient, although an inflation time less than 30 min is recommended.^15^ The severe consequences of aortic occlusion in Zones 1 (supraceliac) and 3 (infrarenal) for placental blood flow added an additional concern in this specific case, and must in general always be included in the cost–benefit analysis.

## 4 CONCLUSION

We present a 31‐year‐old and 11‐week pregnant woman in extremis due to a ruptured splenic artery aneurysm. REBOA allowed temporary haemorrhagic control until definitive, radiological intervention was performed. The use of REBOA has life‐saving potential, and we would argue that this tool should be used more widely, and be on the repertoire for anaesthesiologists.

## AUTHOR CONTRIBUTIONS

Marte Irene Skille Carlsen, Nina Kirksæther Aalberg, Alina Desiree Sandø and Solveig Skrede contributed to the acquisition of case details. Marte Irene Skille Carlsen and Nina Kirksæther Aalberg wrote the first draft of the manuscript. Nina Kirksæther Aalberg supplied the diagnostic images. Marte Irene Skille Carlsen, Nina Kirksæther Aalberg, Sven Erik Gisvold and Oddvar Uleberg critically revised the manuscript. All authors have given final approval of this version to be published.

## FUNDING INFORMATION

Not applicable.

## CONFLICT OF INTEREST

The authors declare that they have no conflict of interest.

## Data Availability

Data sharing is not applicable to this article as no new data were created or analysed in this study.
